# Outlier Detection Method in Linear Regression Based on Sum of Arithmetic Progression

**DOI:** 10.1155/2014/821623

**Published:** 2014-07-10

**Authors:** K. K. L. B. Adikaram, M. A. Hussein, M. Effenberger, T. Becker

**Affiliations:** ^1^Group Bio-Process Analysis Technology, Technische Universität München, Weihenstephaner Steig 20, 85354 Freising, Germany; ^2^Institut für Landtechnik und Tierhaltung, Vöttinger Straße 36, 85354 Freising, Germany; ^3^Computer Unit, Faculty of Agriculture, University of Ruhuna, Mapalana, 81100 Kamburupitiya, Sri Lanka

## Abstract

We introduce a new nonparametric outlier detection method for linear series, which requires no missing or removed data imputation. For an arithmetic progression (a series without outliers) with *n* elements, the ratio (*R*) of the sum of the minimum and the maximum elements and the sum of all elements is always 2/*n* : (0,1]. *R* ≠ 2/*n* always implies the existence of outliers. Usually, *R* < 2/*n* implies that the minimum is an outlier, and *R* > 2/*n* implies that the maximum is an outlier. Based upon this, we derived a new method for identifying significant and nonsignificant outliers, separately. Two different techniques were used to manage missing data and removed outliers: (1) recalculate the terms after (or before) the removed or missing element while maintaining the initial angle in relation to a certain point or (2) transform data into a constant value, which is not affected by missing or removed elements. With a reference element, which was not an outlier, the method detected all outliers from data sets with 6 to 1000 elements containing 50% outliers which deviated by a factor of ±1.0*e* − 2 to ±1.0*e* + 2 from the correct value.

## 1. Introduction

Outlier detection and management of missing data are the two major steps in the data cleaning/cleansing process [1–3]. For achieving a training set, data mining, and statistical analyses, it is very important to have data sets that have no (or as few as possible) outliers and missing values. Except for model-based approaches, outlier detection and replacing of detected outliers or replacing missing values are two separate processes.

The existing outlier detection methods are based on statistical, distance, density, distribution, depth, clustering, angle, and model approaches [1, 4–7]. The nonparametric outlier detection methods are independent of the model. For the data without prior knowledge, nonparametric methods are known as a better solution than the statistical (parametric) methods [8–10]. The most common nonparametric methods are based on distance, density, depth, cluster, angle, and resolution techniques. Among various methods/techniques are least square method (LSM) [4] and the sigma filter [11] which have been used frequently to remove the outliers of linear regression. These methods require data in Gaussian or near Gaussian distribution, which cannot be always guaranteed. If the correct model can be identified, model-based approaches like the Kalman filter [12–14] are suitable for removing and replacing outliers. However, if it is not possible to identify the correct model, the model-based approach is not feasible [15].

In addition to the noise, missing data is another challenge in the data cleaning/cleansing process. Even if the original data set is without missing elements, removing outliers (without replacement) automatically creates a missing data environment. The most common two techniques to recover this situation are (1) filling the missing data with an estimated value (filling) or (2) using the data without missing values (reject missing values). Complete-case analysis (listwise deletion) and available-case analysis (pairwise deletion) are the most common missing data rejection methods [16–18]. The mentioned methods are under the assumption that they yield unbiased results. Among the different missing data filling methods hot deck, cold deck, mean, median,* k*-nearest neighbours, model-based methods, maximum likelihood methods, and multiple imputation are the most common methods [18–22]. Filling methods derive the filling value from the same or other known existing data. If there are a considerable number of outliers, derived data may be biased due to the influence of outliers [23, 24]. Therefore, the best way is to remove all outliers and replace the outliers with a suitable method.

In this paper, we introduce a new nonparametric outlier detection method based on sum of arithmetic progression, which used an indicator 2/*n*, where *n* is the number of terms in the series. The properties used in existing nonparametric methods such as distance, density, depth, cluster, angle, and resolution are domain dependent. In contrast, the value 2/*n*, which we used in our new method, is independent of the domain conditions.

Contrary to the existing nonparametric methods mentioned earlier this work addressed identifying outliers in a dataset that is expected to have linear relation. The method is capable of identifying significant and nonsignificant outliers, separately. Moreover, until all the outliers were removed, the new method requires no missing or removed data imputation. This will eliminate the negative influence due to wrongly filled data points. This is an advantage over the methods, which require filling the removed data points. The outlier detection method we introduced showed its best performances when the significant outliers are in non-Gaussian distribution. This is an advantage over existing methods such as LMS and sigma filter. The method uses a single data point as a reference data point. The reference point is assumed to be nonoutlier. Therefore, accuracy of the outcome is depending on the reference point, especially when locating nonsignificant outliers. If the selected reference point is not an outlier, the method was capable of locating outliers from a data set containing very high rate of outliers, such as 50% outliers.

In this work, data from biogas plants were used for evaluating the new method. Since the biogas process is very sensitive, these data contain a considerable amount of noise even during apparently stable conditions. This provides suitable data set for evaluating our method. We were able to get the best outlier-free macroscale data set which agrees with linear (increasing, decreasing, or constant) regression from selected segments of a data set.

## 2. Methodology

### 2.1. Arithmetic Progression

An arithmetic progression (AP) or arithmetic sequence is a sequence of numbers (ascending, descending, or constant) such that the difference between the successive terms is constant [25]. The *n*th term of a finite AP with *n* elements is given by

(1) $$\begin{array}{c} a_{n}=d\left(n-1\right)+a_{1}, \end{array}$$

where *d* is the common difference of successive members and *a*_1_ is the first element of the series. The sum of the elements of a finite AP with *n* elements is given by

(2) $$\begin{array}{c} S_{n}=\left(\frac{n}{2}\right)*\left(a_{1}+a_{n}\right), \end{array}$$

where *a*_1_ is the first element and *a*_*n*_ is the last element of the series.

Equation (1) is a *f*(*n*) and fulfils the requirements of a line. In other words, finite AP is a straight line. In addition, a straight line is a series without outliers. If there are outliers, the series is not a finite AP. Therefore, any arithmetic series that fulfils the requirements of an AP can be considered a series without outliers. Equation (2) can be represented as

(3) $$\begin{array}{c} \frac{2}{n}=\frac{\left(a_{1}+a_{n}\right)}{S_{n}};\;\infty >n\geq 2,\;\;0<\frac{2}{n}\leq 1. \end{array}$$

For any AP, the right-hand side (RHS) of (3) is always 2/*n*, which is independent of the terms of the series. In other words, if there are no outliers, the value (*a*_1_ + *a*_*n*_)/*S*_*n*_ will always be equal to 2/*n*. If the RHS of (3) is not 2/*n*, it always implies that the series contains outliers. Therefore, the value 2/*n* can be used as a global indicator to identify any AP with outliers.

Since we use the relation of AP, we define that elements lying on or between two lines (linear border) are nonoutliers, and others are outliers. When the distance between two lines is zero, they represent a single line. In relation to the method presented in this paper, the term nonoutlier implies an element that lies within a certain linear border, and the term outlier implies an element that does not lie within the linear border.

Primary investigations showed that the method is capable of not only indicating the existence of outliers but also locating the outlier. (*a*_1_ + *a*_*n*_)/*S*_*n*_ < 2/*n* indicates that the maximum element is the outlier. (*a*_1_ + *a*_*n*_)/*S*_*n*_ < 2/*n* indicates that the minimum element is the outlier. However, (*a*_1_ + *a*_*n*_)/*S*_*n*_ = 2/*n* does not imply that the series is free of outliers. Furthermore, primary investigations showed that the method is capable of locating both large and small outliers.  Table 1 shows sample calculations for illustrating the relation between 2/*n* and (*a*_1_ + *a*_*n*_)/*S*_*n*_.

As a principle, the relation of (3) is capable of identifying and locating the outliers. However, we found seven drawbacks, which made relation (3) unusable for identifying outliers in actual data. In Sections 2.1 to 2.7, we address the challenges for making the relation usable.

### 2.2. Challenge 1: Notation of the Equation

The symbols used in (3), especially *a*_1_, *a*_*n*_, create a logical barrier. For example, if there are outliers, the minimum and the maximum can be other elements rather than *a*_1_, *a*_*n*_. Therefore, it is necessary to use meaningful symbols that reflect the purpose of the method. The first and the last elements are either the minimum or the maximum. Therefore, it is possible to replace *a*_1_ and *a*_*n*_ by the minimum (*a*_min⁡_) and the maximum (*a*_max⁡_) of the series. Then (3) can be represented as

(4) $$\begin{array}{c} \frac{2}{n}=\frac{\left(a_{\min}+a_{\max}\right)}{S_{n}}. \end{array}$$

Since the RHS of (4) consists of minimum, maximum, and sum of the series, RHS was named MMS with the meaning of minimum, maximum, and sum:

(5) $$\begin{array}{c} \text{MMS}=\frac{\left(a_{\min}+a_{\max}\right)}{S_{n}}. \end{array}$$

### 2.3. Challenge 2: Set a Range for the Outlier Detection Criterion

**Figure 1:**
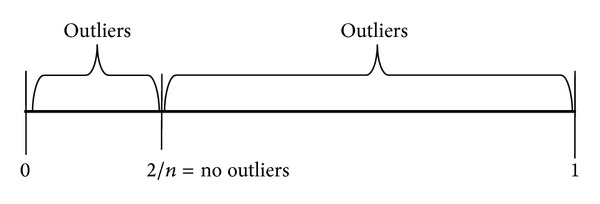
Distribution of criteria range (0, 1].

According to (3), outlier detection criterion is 2/*n* and can be used to check the elements that exactly agree with a line (Figure 1). To identify elements in a certain range, it is necessary to have a criteria range rather than a single value 2/*n*.

The left-hand side of (4) is the ratio 2 :* n* and named as *R*_*w*_ by adding a weight “*w*” to “*R*.” Then,

(6) $$\begin{array}{c} R_{w}=\frac{2}{n}\;+w;\;0\leq w\leq 1-\frac{2}{n}. \end{array}$$

The status *w* = 0 (*R*_0_) represents a single line, and *w* > 0 represents a line with a certain width (linear border). The outlier criteria range is a range with both floor (0) and ceiling (1), and standardization is not required. This is an additional advantage over the most common average, variance, and slandered deviation based approaches, which require a separate standardization process.

### 2.4. Challenge 3: Influence of Negative Values

Due to negative values, the numerator or both the numerator and the denominator of RHS of (5) can be 0 (e.g., −4, − 1, 0, 1, 4), even without outliers. When there are outliers, RHS of (5) can be negative, which cannot be accepted as valid values for 2/*n*, 0 < 2/*n* ≤ 1, must always hold.

Subtracting the first element (*a*_*i*_new_ = *a*_*i*_ − *a*_min⁡_) from each element of any AP creates a new transformed AP where *a*_min⁡_ = 0 and guarantees a series without negative values. From (5) and *a*_*i*_new_ = *a*_*i*_ − *a*_min⁡_, (7) is derived, which is more robust. Another advantage of (7) is that it performs the transformation, automatically:

(7) $$\begin{array}{c} \text{MMS}=\frac{\left(\left(a_{\min}-a_{\min}\right)+\left(a_{\max}-a_{\min}\right)\right)}{\sum_{i=1}^{n}\left(a_{i}-a_{\min}\right)},\\ \text{MMS}=\frac{\left(a_{\max}-a_{\min}\right)}{\left(S_{n}-a_{\min}*n\right)}. \end{array}$$

### 2.5. Challenge 4: Uneven Distribution of Criteria Range

The ranges (0,2/*n*) and (2/*n*, 1] are to identify outliers, which are minimums and maximums, respectively (Figure 1). When *n* → *∞* and *R*_0_ → 0, then *R*_*w*_ : (0, 1] is not equally distributed, which provides a large range for maximum outliers and a small range for minimum outliers. This is a problem when locating minimum outliers.

To solve this, we used the idea of complement. For any series, this will convert the maximum value into the minimum, the minimum value into the maximum, and intermediate values into their complements. Most importantly, now the minimum value represents the maximum value of the original series and vice versa, while still representing the original series. The complement of an element in a series can be defined as *a*_*i*_*c*_ = (*a*_max⁡_ + *a*_min⁡⁡_) − *a*_*i*_. From (5) and *a*_*i*_*c*_ = (*a*_max⁡_ + *a*_min⁡⁡_) − *a*_*i*_ this gives

(8) $$\begin{array}{c} \text{MMS}=\frac{\left(\left(a_{\max}+a_{\min}-a_{\max}\right)+\left(a_{\max}+a_{\min}-a_{\min}\right)\right)}{\sum_{i=1}^{n}\left(a_{\max}+a_{\min}-a_{i}\right)},\\ \text{MMS}=\frac{\left(\left(a_{\min}\right)+\left(a_{\max}\right)\right)}{\sum_{i=1}^{n}\left(a_{\max}+a_{\min}-a_{i}\right)}. \end{array}$$

Apply *a*_*i*_new_ = *a*_*i*_ − *a*_min⁡_ (to remove effect from negative values):

(9) $$\begin{array}{c} \text{MMS}=\frac{\left(\left(a_{\min}-a_{\min}\right)+\left(a_{\max}-a_{\min}\right)\right)}{\sum_{i=1}^{n}\left(\left(a_{\max}-a_{\min}\right)+\left(a_{\min}-a_{\min}\right)-\left(a_{i}-a_{\min}\right)\right)},\\ \text{MMS}=\frac{\left(a_{\max}-a_{\min}\right)}{\sum_{i=1}^{n}\left(a_{\max}-a_{i}\right)},\\ \text{MMS}=\frac{\left(a_{\max}-a_{\min}\right)}{\left(a_{\max}*n-S_{n}\right)}. \end{array}$$

**Figure 2:**
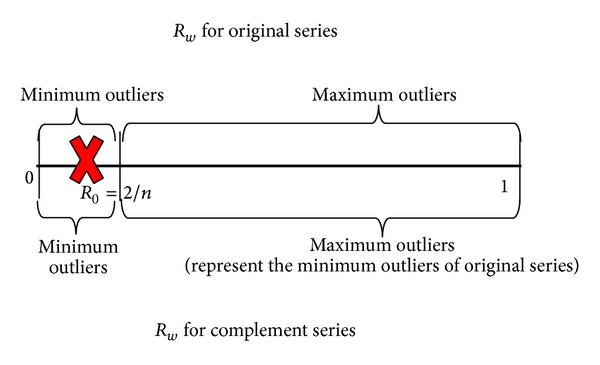
Range of *R*_*w*_ for original series and complement of original series.

Consequently, the range *R*_0_ > 2/*n* represents the range for minimum outliers related to the original series and vice versa (Figure 2), and it is possible to ignore the range (0,2/*n*). In addition, (9) automatically performs the transformation.

Now there are two equations for MMS, (7) and (9), to check whether the maximum or the minimum of the series is an outlier. We named the two versions of MMS as MMS_max⁡_ (10) and MMS_min⁡_ (11)

(10) $$\begin{array}{c} {\text{MMS}}_{\max}=\frac{\left(a_{\max}-a_{\min}\right)}{\left(S_{n}-a_{\min}*n\right)}, \end{array}$$

(11) $$\begin{array}{c} {\text{MMS}}_{\min}=\frac{\left(a_{\max}-a_{\min}\right)}{\left(a_{\max}*n-S_{n}\right)}. \end{array}$$

The following equation shows the overview of the MMS process:

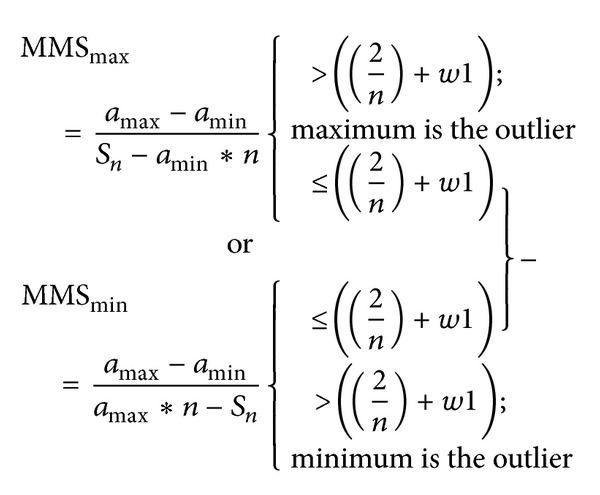
(12)
and Table 2 shows sample calculations using (10) and (11) for the same data sets in Table 1.

### 2.6. Challenge 5: How to Deal with Removed Outliers/Missing Values

**Figure 3:**
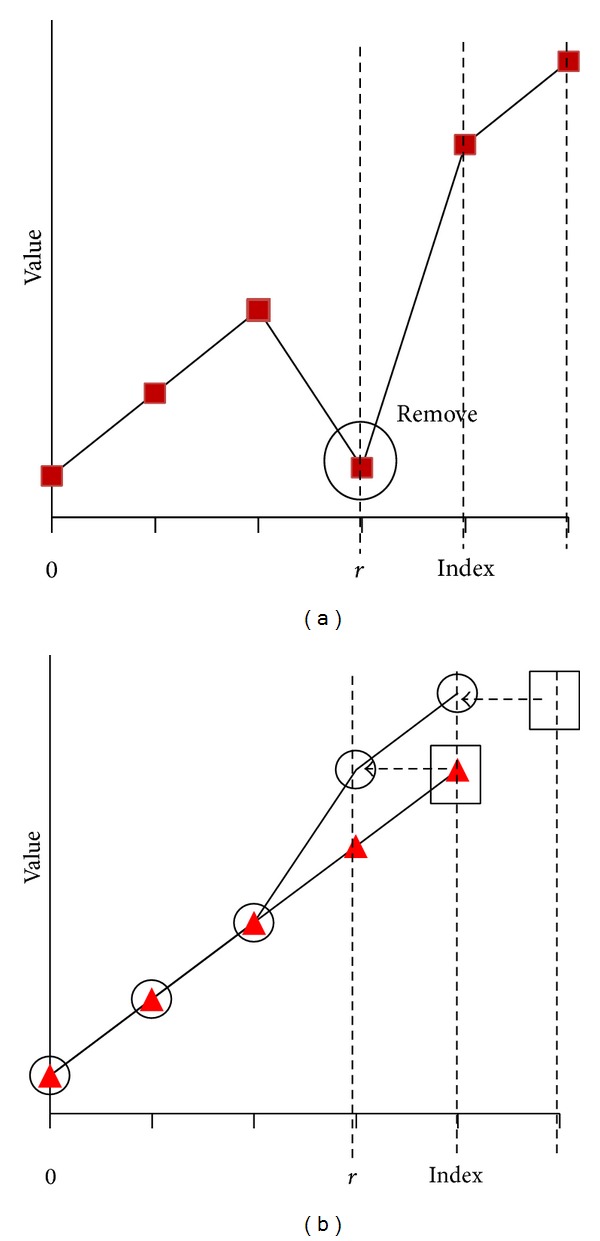
(a) Data set with an outlier at index* r*. (b) Value autotransformation effect after removing the outlier at index* r* without replacement, where circle corresponds to elements after removing the outlier, red triangle corresponds to expected (correct) elements, and square corresponds to initial values of the shifted elements after removing the outlier.

In a series, there can be initial missing values. In addition, if there is no replacement after removing an outlier it also creates a missing value environment. If there is no filling, it would transform the elements after the element is removed into another value and destroy the original relationship of elements (Figure 3). These transformed values become outliers in relation to the original data. Therefore, for using the relation of AP, it is compulsory to maintain the original relation of the data even after removing an outlier. Thus, any rejection technique is not feasible. To maintain the original relation, one possible way is replacing the missing value. However, the data we are considering contain a considerable amount of outliers. Therefore, we cannot guarantee that an element derived from existing elements is not an outlier.

To overcome this problem, we considered two different options: (1) recalculate only the data points after (or before) the removed or missing element, thereby maintaining the initial angle in relation to a certain point or (2) transform the elements into a new series where the missing value has no effect.

#### 2.6.1. Recalculate the Data Points after (or before) Removed and Missing Elements

If there is a missing element, the next elements will be shifted horizontally and transformed into wrong values in relation to the current index of the elements (Figure 3). However, angular shifting will not introduce such an error (Figure 3).

**Figure 4:**
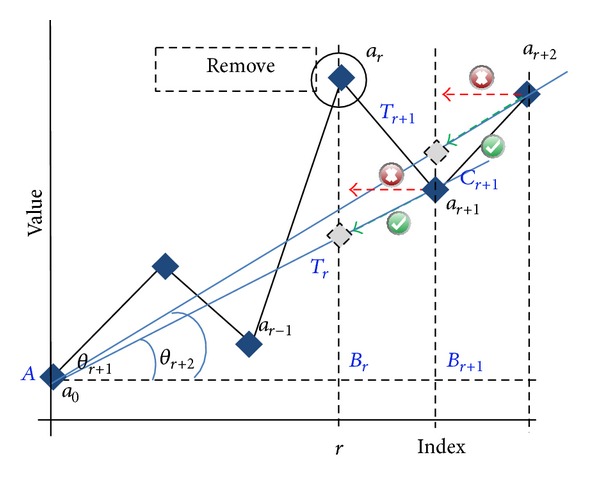
Solution for value autotransformation phenomenon. Use angular shifting instead of horizontal shift, where (×) corresponds to horizontal shift and (*✓*) corresponds to angular shift.

In Figure 4, the plot consists of elements *a*_0_ to *a*_*r*+1_ (*r* ∈ *R* ^+^), and element *a*_*r*_ at *r* needed to be removed. After removing element *r*, element *r* + 1 becomes element *r*, element *r* + 2 becomes element *r* + 1, and so on. However, shifting while maintaining the same angle with respect to a certain reference element (e.g., the first element), the same form of the series can be maintained. Equation (13) shows the new value after angular shifting. We used this technique with MMS algorithm to recalculate the series after (or before) missing values or removed elements:

(13) $$\begin{array}{c} B_{r}T_{r}=\left(\frac{B_{r+1}C_{r+1}}{AB_{r+1}}\right)*AB_{r}=\left(\frac{\left(a_{r+1}-a_{0}\right)}{\left(r+1\right)}\right)*r,\\ {\left(a_{r+1}\right)}_{\text{new}}=a_{o}+B_{r}T_{r}. \end{array}$$

#### 2.6.2. Transformation of Data to a Constant Value

A series with a constant value (*y* = *c* form, where *c* is a constant) is a series that has no effect of missing values. Because of that, if it is possible to transform any linear series to *y* = *c* form, the transformed series is free of any effect of missing values. After that, the transformed series can be used for outlier detection.

**Figure 5:**
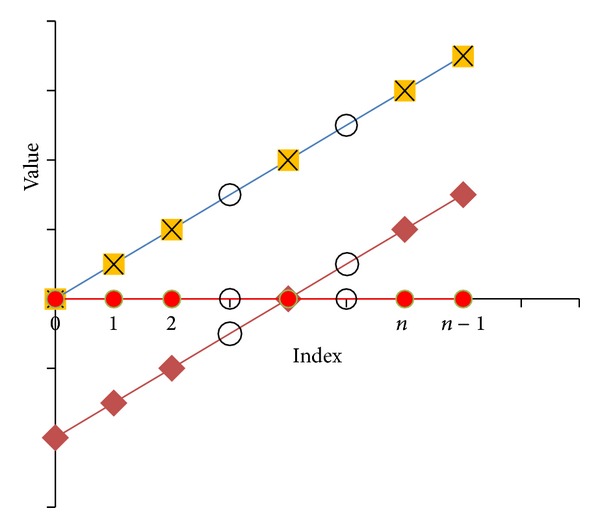
Transformation of data to a constant value to overcome the missing data problem, where red diamond corresponds to *y* = *f*(*x*) form, yellow square corresponds to *y* ^*T*^ = *f*(*x*) − *f*(*x*_0_) form, cross corresponds to *y*′ = *m*∗*x*_*i*_ form (*m* = ∑_*i*=0_^*n*^ *y*_*i*_^*T*^/∑_*i*=0_^*n*^ *x*_*i*_^*T*^), red circle corresponds to *y* ^*TT*^ = *y* ^*T*^ − *y*′, and circle corresponds to missing values.

If *y* ^*T*^ is a linear series, where *y*_*k*_^*T*^ = *y*_*k*_ − *y*_1_, *x*_*k*_^*T*^ = *x*_*k*_ − *x*_1_, *x*_*k*_ is the initial index of elements and *y*_*k*_ is the *k*th element of the series, *k* = 1,2,…, *n*. The gradient of the line (*m*) is given by ∑_*i*=1_^*n*^ *y*_*k*_/∑_*i*=1_^*n*^ *x*_*k*_. If one element (e.g., the first element) is (0,0), this relation is always true even with missing values. The element (0,0) can be considered as the reference element. The *y* ^*T*^ is a series with first element (0,0) and *m* that can be calculated even with missing values. Also, it is possible to derive a new series as *y*′ where *y*_*k*_ = *x*_*k*_∗*m*. If there are no outliers, both *y* ^*T*^ and *y*′ coincide and *y* ^*T*^ − *y*′ = 0. If *y* ^*TT*^ = *y* ^*T*^ − *y*′, *y* ^*TT*^ is in the form of *y* = *c* without any influence from missing values. Therefore, this is another method to overcome missing values without replacing them (Figure 5).

### 2.7. Challenge 6: Locate Outliers That Are Neither the Maximum Nor the Minimum of the Series

When the outlier is neither the maximum nor the minimum, MMS is unable to locate the outlier (Table 3). We named this phenomenon as “Bad Detection.” When *R*_*w*_ reaches “Bad Detection Level,” MMS cannot be applied. To overcome this situation, we introduced an improved version of MMS as enhanced MMS (EMMS) based on the missing data imputation technique in Section 2.6.2.

EMMS is expressed as

(14) $$\begin{array}{c} {\text{EMMS}}_{\max}=\frac{\left(a_{\max}^{TT}-a_{\min}^{TT}\right)}{\left(S_{n}^{TT}-a_{\min}^{TT}*n\right)};\;a_{\max}^{TT}\langle \;\rangle 0, \end{array}$$

(15) $$\begin{array}{c} {\text{EMMS}}_{\min}=\frac{\left(a_{\max}^{TT}-a_{\min}^{TT}\right)}{\left(a_{\max}^{TT}*n-\;S_{n}^{TT}\right)};\;a_{\max}^{TT}\langle \;\rangle 0, \end{array}$$

where *a*_*k*_^*TT*^ = |*a*_*k*_^*T*^ − *x*_*k*_(*Ga* ^*T*^/*Gx*)|, *a*_*k*_^*T*^ = *a*_*k*_ − *a*_0_, *x*_*k*_ is the index of data, *a*_*k*_ is the *k*th term of the series, *k* = 0,1,…, *n* − 1, *n* is the number of elements in current window, *Ga* ^*T*^ = ∑_*k*=0_^*n*−1^ *a*_*k*_^*T*^, *Gx* = ∑_*k*=0_^*n*−1^ *X*_*k*_, and *S*_*n*_^*TT*^ = ∑_*k*=0_^*n*−1^ *a*_*k*_^*TT*^〈 〉0.

Always the term *a* ^*TT*^ > 0. Thus, the term *a*_min⁡_^*TT*^ = 0. Then (14) and (15) are simplified as

(16) $$\begin{array}{c} {\text{EMMS}}_{\max}=\frac{a_{\max}^{TT}}{S_{n}^{TT}};\;a_{\max}^{TT}\langle \;\rangle 0, \end{array}$$

(17) $$\begin{array}{c} {\text{EMMS}}_{\min}=\frac{a_{\max}^{TT}}{\left(a_{\max}^{TT}*n-S_{n}^{TT}\right)};\;a_{\max}^{TT}\langle \;\rangle 0. \end{array}$$

If there are outliers, EMMS_min⁡_ > 2/*n* or EMMS_max⁡_ > 2/*n* and the greater value represents the outlier.  Table 4 shows an example calculation of EMMS and the following equation shows an overview of EMMS process:

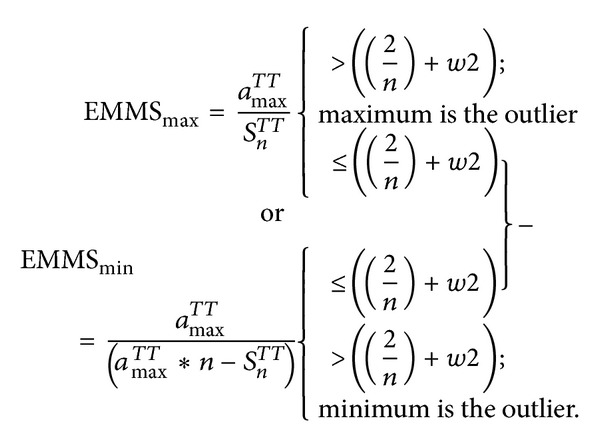
(18)


However, EMMS uses derived information from existing data. If there are biased values, it may lead to biased information. Because of that, direct application of EMMS is not a good practice. Hence, significant outliers should be removed first using MMS, before applying EMMS.

### 2.8. Challenge 7: Determining of Outlier Detection Criteria (*R*_*w*_)

The value *R*_*w*_ is the factor that determines the outliers, when *w* = 0 (*R*_0_ = 2/*n*) represents exactly a line and *w* > 0 represents a linear border with certain width. In this section, we propose several possible methods that can be used to determine the outlier detection criteria.

#### 2.8.1. Express the Value “*w*” as *f*(1/*n*)

If the value* w* is *f*(1/*n*) then *w* = 2∗*k*/*n*; *k* ≤ (*n*/2) − 1; and *k* ∈ *R* ^+^. Then *R*_*w*_ = 2/*n* + 2∗*k*/*n*:

(19) $$\begin{array}{c} R_{w}=\frac{2}{n}*\left(1+k\right),\\ \frac{R_{w}}{R_{0}}=1+k\left(=\text{constant}\right). \end{array}$$

**Figure 6:**
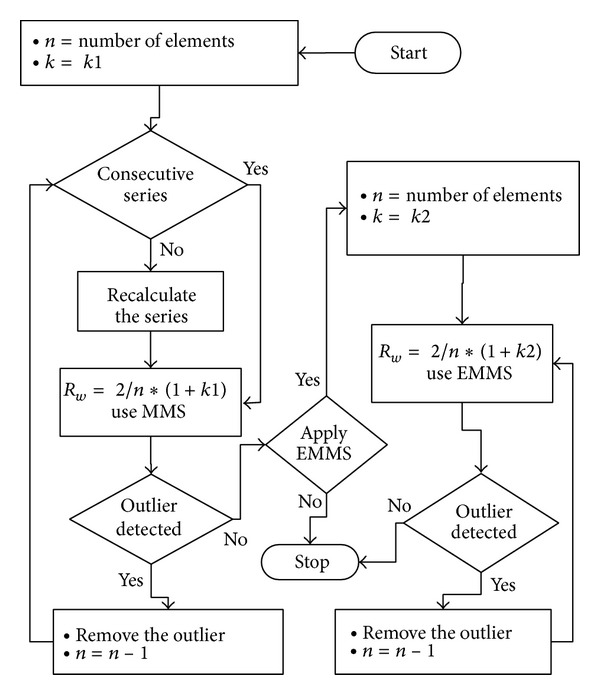
Implementation of MMS and EMMS. Initially algorithm checks for the significant outliers using MMS. After removing all significant outliers, then remove the nonsignificant outliers using EMMS. There is no removed data imputation in relation to both MMS and EMMS.

When the MMS or the EMMS is greater than *R*_*w*_ of (19), this implies the existence of outliers. Because *R*_*w*_/*R*_0_ is constant and gives standards to *R*_*w*_, determination of* k* still depends on the knowledge of the domain.  Figure 6 shows an algorithm based on this technique.

#### 2.8.2. When the First and the Last Items Are Nonoutliers

In the total process, the “Bad Detection level” is the most important criteria. If* R*_*w*_ of MMS is less than the “Bad Detection Level” it is possible to identify nonoutliers as outliers as mentioned in Section 2.7. If there is preknowledge about outliers, it is possible to use a safe value for MMS. Otherwise, there is no 100% guarantee on “Bad Detection Level.”

**Figure 7:**
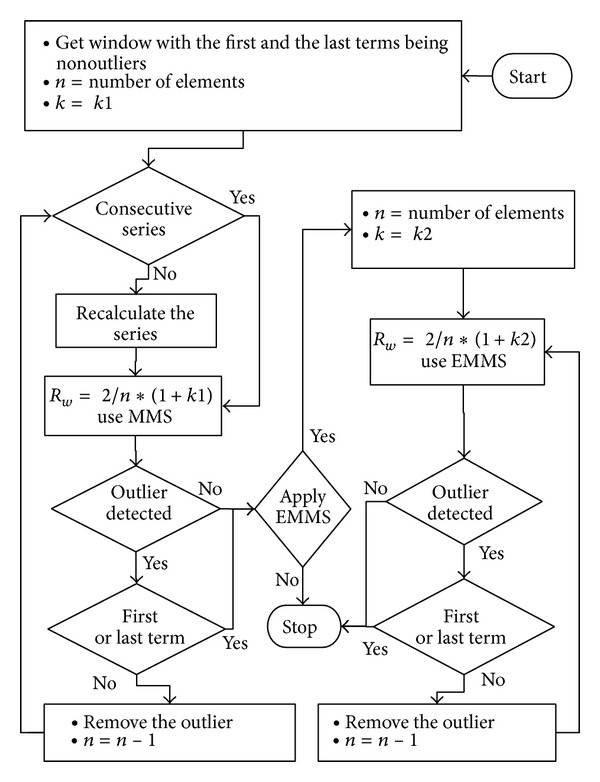
Outlier detection method including the “Bad Detection Level” detection technique. The first and the last data points of the window must be nonoutliers. If the first or the last element was identified as an outlier, it will become a contradictory situation. Thus, this point can be considered as the terminating point of MMS and EMMS.

However, when the first and the last elements are not outliers, the “Bad Detection Level” can be detected automatically. If the first or the last element was identified as an outlier, it will become a contradictory situation. Thus, this point can be considered as the terminating point of MMS and EMMS. The decision diagram elaborated in Figure 7 expresses the new outlier detection method including the “Bad Detection Level” detection technique.

### 2.9. Validate the Method

We implemented the MMS (with recalculation after an outlier is removed) and EMMS with C++ and conducted the validation process. For the recalculation process, the existing first element of the window was the reference element and always used the original value of the element (not the current updated value of the element). To validate the method, we used artificial data sets of different sizes (10 to 1000) of a line representing increasing, decreasing, and constant line. Then 50% of items of those data sets were replaced with very small and very large outliers (±1.0*e* − 2 to ±1.0*e* + 2 times of correct value). We checked the data sets for all the environment combinations shown in Table 5. The outlier detection criteria were determined based on (19). For all data sets, the same* k* value was used (for MMS, *k* = 0.5, and for EMMS, *k* = 0.01). Then the percentage of correctly and falsely detected nonoutliers in relation to the number of actual nonoutliers and the percentage of correctly and falsely detected outliers from the total number of outliers (small and large outliers) were determined.

### 2.10. Evaluation Using Real Data

To check the best linear fitting identification capability, the algorithm was tested using several real data sets which were automatically recorded with a frequency of twelve data points per day (i.e., every other hour) from a biogas plant, over a period of seven months. Among the different parameters, we selected the H_2_ content measured in ppm, which we expected to maintain linear behaviour during stable operation. We selected seven segments of different size for evaluating the algorithm. In some data sets, there were initial missing elements. We set the *R*_*w*_ for MMS and EMMS by analysing the first and the third data sets. For the recalculation process, the existing first element of the window was the reference element, and we always used the original value of the elements (not the current updated value of the element). Then the percentage of correctly falsely detected nonoutliers in relation to the total number of nonoutliers and the percentage of correctly and falsely detected outliers from the total number of outliers (small and large outliers) were determined.

We decided to use the LSM, Sigma filter, and Grubb's test [26–29] also known as maximum normed residual test or “extreme studentized deviate” (ESD) test to compare our results. We selected Grubb's test since it has nearly the same formulation as our method. We checked all the biogas data using abovementioned methods. We used each of the data segments as a single window. First, we checked the ability of each method to identify the general trend of the series. Then, we checked the amount of correctly and falsely detected outliers and nonoutliers for each method in relation to the general trend.

## 3. Results and Discussion

**Figure 8:**
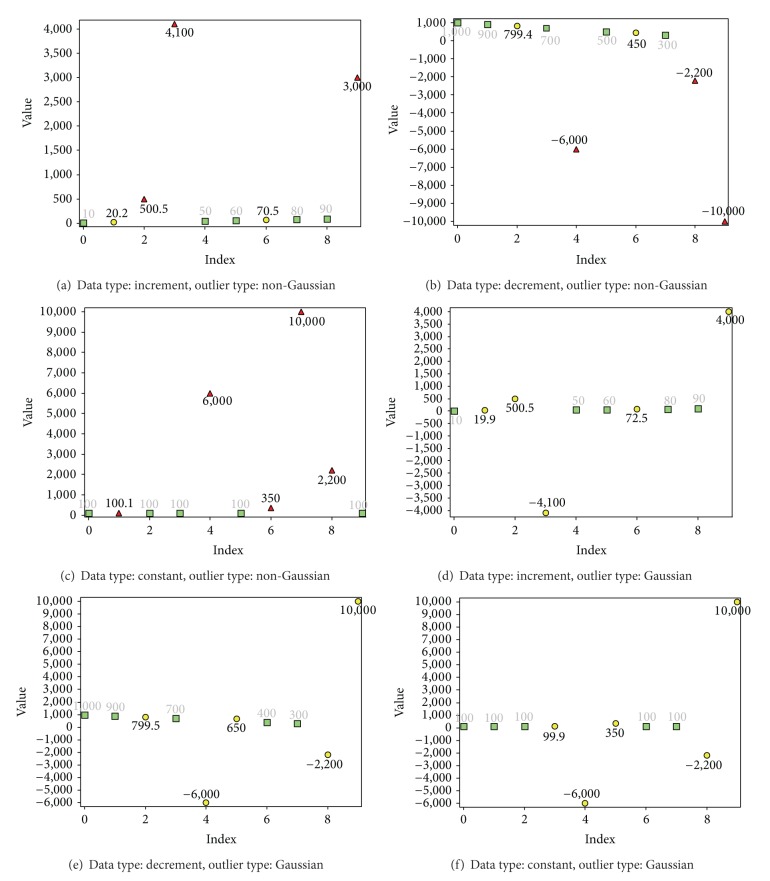
Outlier detection from data sets with ten elements. The first element is the reference element, which is not an outlier, where red triangle corresponds to outliers detected by MMS, yellow circle corresponds to outliers detected by EMMS, and green square corresponds to nonoutliers. Value of* k* for MMS and EMMS is 0.5 and 0.01, respectively. When the reference (first) element is not an outlier, the new method is capable of locating all outliers. When the outliers are Gaussian, MMS automatically becomes inactive (now no significant outliers) ((d), (e), (f)).

**Figure 9:**
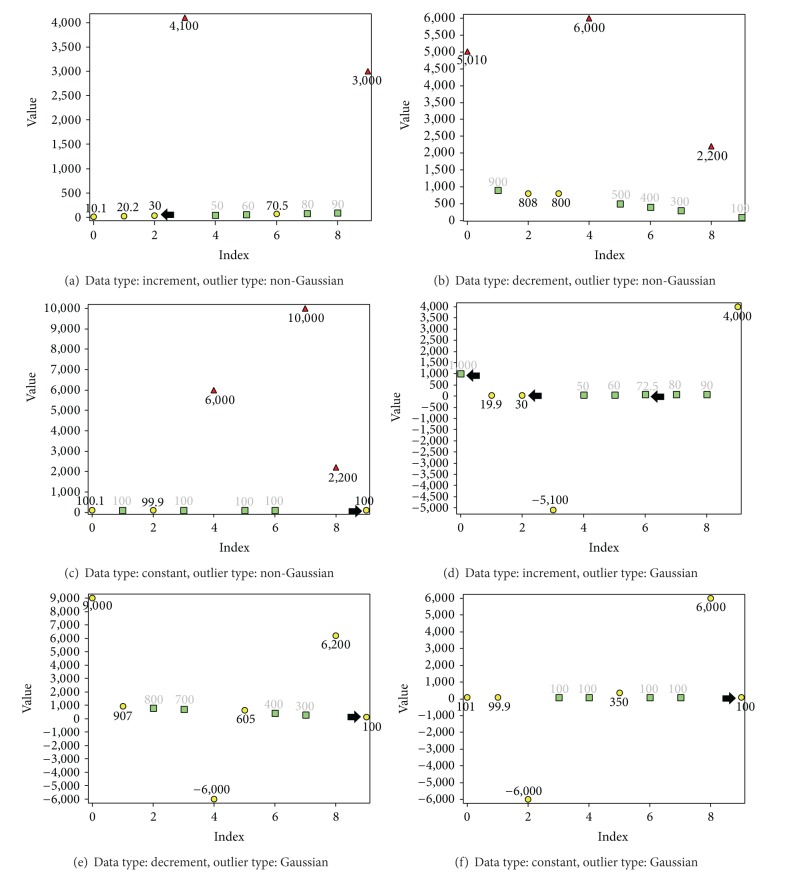
Outlier detection from data sets with ten elements. The first element is the reference element, which is an outlier, where red triangle corresponds to outliers detected by MMS, yellow circle corresponds to outliers detected by EMMS, green square corresponds to nonoutliers, and black arrow corresponds to wrong detections. Value of* k* for MMS and EMMS is 0.5 and 0.01, respectively. When the reference (first) element is an outlier and outliers are non-Gaussian, the new method identifies only the significant outliers ((a), (b), (c)). When the outliers are Gaussian, MMS automatically becomes inactive (now no significant outliers) ((d), (e), (f)).

Results related to validation show that when the reference element (the first element) was not an outlier, the algorithm was capable of identifying all outliers with 0% error despite of the type of outliers (Gaussian or non-Gaussian) (Figure 8). If the outliers were Gaussian, there were no significant outliers and MMS automatically became inactive (Figures 8(d), 8(e), and 8(f)). When the first few elements were outliers and outliers were non-Gaussian, MMS detected the significant outliers correctly (Figures 9(a), 9(b), and 9(c)). However, EMMS was unable to locate the nonsignificant outliers, when the first element for EMMS was an outlier (Figures 9(a) and 9(c)). If the reference element for EMMS was not an outlier, it was still possible to achieve correct results (Figure 9(b)). Though it was impossible to locate all nonoutliers, the detected nonoutliers were 100% correct detections. These values can be used to estimate the other values using methods like LSM since now all the existing data are cleaned. In general, it is fair to state that (1) when the reference element is not an outlier, the method is capable of identifying all outliers and (2) when the first few elements of the series are outliers and the outliers are non-Gaussian, the method is capable of identifying only the significant outliers and part of correct elements.

When the first few elements (reference elements for both MMS and EMMS) were outliers and the outlier distribution was Gaussian, outlier detection was poor (Figures 9(d), 9(e), and 9(f)). Due to the Gaussian distribution of outliers, MMS was inactive and it was not possible to identify the large outliers. Most importantly, the results highlighted the importance of the reference element. If the reference element for MMS and EMMS was not an outlier, it guaranteed good results despite of other factors.

In the methodology, we derived the method based on the first element. However, it is also possible to use any other element as reference point and modify the method. We considered the simplest situation, where the first element is not an outlier. Therefore, if it is possible to segment the data excluding extreme outliers at the beginning, it provides accurate outlier detection. Another possibility is to replace the first element with an already known element. This leads to another possibility for applying the method: if we know only a single correct element, the use of that element as reference element and of the modified method according to the reference element can yield very accurate results.

Some model-based approaches demand a trained data set for correct output. In contrast, this method requires only one correct element to produce a correct output. In addition, it is possible to use multiple reference points and consider the best fitting. For example, (a) consider each point in first* x*% (e.g., 10%) of data points as reference point and (b) consider all data points as the reference point. Furthermore, it is important to distinguish the purpose of MMS and EMMS. MMS removes only the significant outliers, while EMMS removes nonsignificant outliers. Depending on the requirement, MMS or/and EMMS can be used to remove outliers.

**Figure 10:**
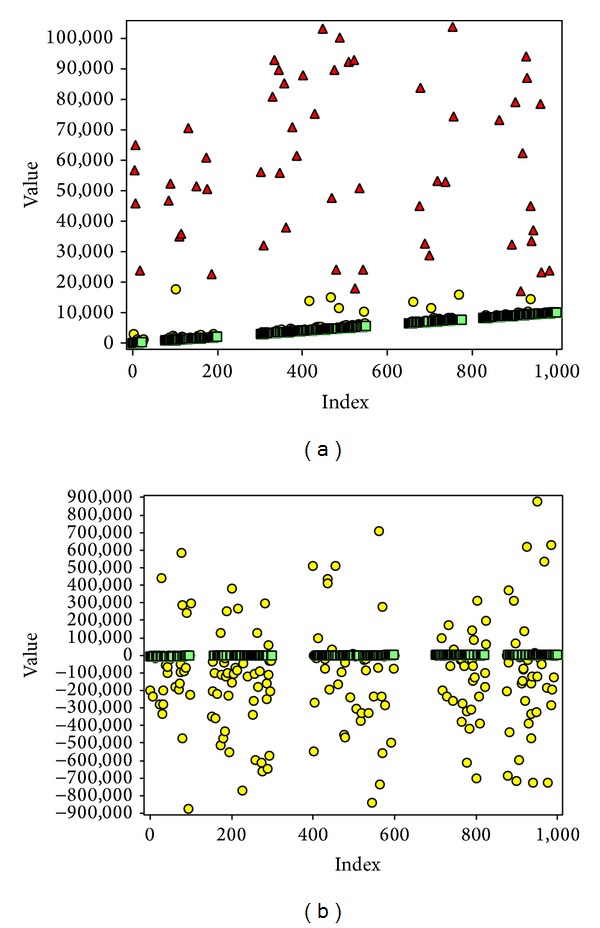
Two artificial data samples with 1000 elements each, including 50, 100, 100, and 50 (total 300) missing value regions. The first element is the reference element, which is not an outlier, where (a) corresponds to a data set with outliers in non-Gaussian, (b) corresponds to a data set with outliers in nearly Gaussian, red triangle corresponds to outliers detected by MMS, yellow circle corresponds to outliers detected by EMMS, and green square corresponds to nonoutliers. The value of* k* for MMS and EMMS is 0.5 and 0.01, respectively. The new method was able to identify all the elements related to the line with 0% error.

The results show that the new method is a good solution for managing missing values.  Figure 10 shows two data sets with 1000 elements each. Each data set consists of 50, 100, 100, and 50 (total 300) missing value regions. When the first element was not an outlier, the new method was able to identify all the elements related to the line with 0% error.

In real world, it is not possible to find nonoutliers that exactly agree with linear regression. Therefore, 100% accuracy is inapplicable. However, it is very important to have a significant outlier-free data set. The new method guaranteed a significant outlier-free data set when the outliers were non-Gaussian. Furthermore, in real world situations, data/outliers are not always in Gaussian distribution. Due to that, we hope the new method can be applied to the majority of outlier detection applications. Our new method is an effective solution for most common LSM and sigma filter need Gaussian outliers. Some methods like sigma filter cannot be applied directly to a certain data segment, and further segmentation (windowing) is required for better results. In contrast, the new method is capable of locating nonoutliers automatically in increment, decrement, or constant form, regardless of the size of the window.

**Figure 11:**
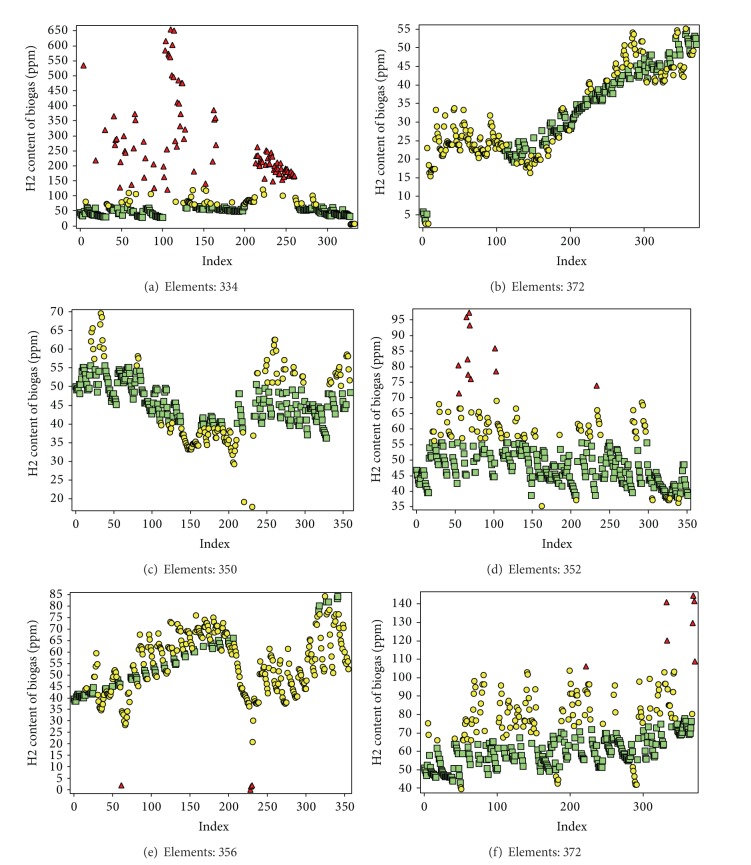
Results related to real biogas data with different size of data sets. The first element is the reference element, which is assumed not to be an outlier. Results showed that the algorithm clearly identifies three regions as significant outliers (outliers from MMS), nonsignificant outliers (outliers from EMMS), and nonoutliers within each data segment. Most importantly, all the nonoutliers lied within a linear border, where red triangle corresponds to outliers detected by MMS, yellow circle corresponds to outliers detected by EMMS, and green square corresponds to nonoutliers. The value of* k* for MMS and EMMS is 0.2 and 0.1, respectively.

Results related to biogas data proved the abovementioned idea and showed that the algorithm clearly identifies three regions as significant outliers (outliers from MMS), nonsignificant outliers (outliers from EMMS), and nonoutliers within a data segment (Figure 11). In addition, the results showed that the nonoutliers follow a linear path. Furthermore, the width of the regions can be tuned by changing the relevant *R*_*w*_ values. Figure 11 shows some selected results of biogas data for a* k* value of 0.2 for MMS and a* k* value of 0.1 for EMMS.

One of the interesting observations was the ability of the algorithm to continue linear detection even with the noncontinuous clusters (Figures 11(b) and 11(e)). In all data segments, there occurred no false detection (there were no outliers in nonoutlier regions and vice versa). Most importantly, the new method required no further windowing and nonoutliers were detected independent of the window size.

When the general trend was constant and elements were in Gaussian distribution, the Sigma filter and LSM were able to identify the linear trend. However, for series with biased elements, both methods failed to identify the general trend. When the general trend was increment or decrement, the Sigma filter failed to identify the general trend (a further segment would give better result, but we used the whole window). The new method was capable of locating 4% to 45% of elements as outliers with 0% error. Grubbs' test was capable of identifying very small amount of elements as outliers (0%–17%), even with the significance level of 0.05. However, all outliers were significant and no wrong detections were reported.

## 4. Conclusions and Outlook

This paper introduced a new outlier detection method using the relation of the sum of the elements of an arithmetic progression. The results of this work prove that the new method is a robust solution for outlier detection in a data set with missing elements. The method is capable of identifying both significant and nonsignificant outliers, when the first value of the data set is not an outlier. Most importantly, the method is a solution for identifying significant outliers in a series with outliers in non-Gaussian distribution. In addition, the outlier detection is nonparametric, has floor and ceiling values, and does not require standardization. When the reference elements are unknown, the method can be used with multiple reference elements to gain optimal output.

If the frequency of the data is sufficient, any nonlinear relation can be represented as a combination of straight lines. Therefore, by using a suitable segmentation technique, it is possible to identify outliers in any data series. This will allow for detecting outliers in a process-oriented data set. Therefore, to bring a data series into a form that is suitable for our method, an intelligent segmentation technique is necessary.

## Figures and Tables

**Table 1:** Sample calculations for illustrating the relation between 2/*n* and (*a*_1_ + *a*_*n*_)/*S*_*n*_.

*a*_*n*_	Data set 1	Data set 2	Data set 3	Data set 4	Data set 5
*a*_1_	100	100	100	99.99	1
*a*_2_	101	101	101	101	101
*a*_3_	102	102	102	102	102
*a*_4_	103	103	103	103	103
*a*_5_	104	104.01	204	104	104

(*a*_1_ + *a*_5_)/*S*_5_	0.4	0.40001	0.498	0.399	0.255
2/*n*	0.4	0.4	0.4	0.4	0.4
Outlier?	—	Yes-*a*_5_	Yes-*a*_5_	Yes-*a*_1_	Yes-*a*_1_

**Table 2:** Sample calculations for illustrating the relation between 2/*n* and MMS_max_ and MMS_min_.

*a*_*n*_	Data set 1	Data set 2	Data set 3	Data set 4	Data set 5
*a*_1_	100	100	100	99.99	1
*a*_2_	101	101	101	101	101
*a*_3_	102	102	102	102	102
*a*_4_	103	103	103	103	103
*a*_5_	104	104.01	204	104	104

MMS (Max)	0.4	**0.401**	**0.945**	0.399	0.254
MMS (Min)	0.4	0.399	0.254	**0.401**	**0.945**
2/*n*	0.4	0.4	0.4	0.4	0.4
Outlier?	—	**Yes-**Max	**Yes-**Max	**Yes-**Min	**Yes-**Min

**Table 3:** “Bad Detection” identified wrong (minimum) element as the outlier.

*a*_*n*_	Data set 6		
*a*_1_	100		
*a*_2_	101	MMS_max_	0.377
*a*_3_	102	MMS_min_	0.425
*a*_4_	103.6	2/*n*	0.4
*a*_5_	104	Outlier?	Yes-Min

**Table 4:** EMMS for identifying an outlier.

*X*(*n* − 1)	*y*(*a*_*n*_)	*y* ^*T*^(*a*_*n*_ − *a*_1_)	*y* ^*TT*^(|*y* ^*T*^ − *x*_*n*_(*G*_*y*_^*T*^/*G*_*x*_)|)
0	100	0.000	0
1	101	1.000	0.06
2	102	2.000	0.12
3	**103.6**	3.600	**0.42**
4	104	4.000	0.24

*G*_*x*_ = 2		*G*_*y*_^*T*^ = 2.120	

		EMMS (Max)	**0.500**
		EMMS (Min)	0.333
		2/*n*	0.4
		Outlier?	Yes-Max

**Table 5:** Different environments used to validate the new method.

Type of the original dataset	Number of elements	Type of outliers	Reference (first) element is an outlier?	Initial missing values?
Increment, constant, and decrement.	10 to 1000	Non-Gaussian, Gaussian	Yes, no	Yes, no
